# The Genome of Chelonid Herpesvirus 5 Harbors Atypical Genes

**DOI:** 10.1371/journal.pone.0046623

**Published:** 2012-10-02

**Authors:** Mathias Ackermann, Maxim Koriabine, Fabienne Hartmann-Fritsch, Pieter J. de Jong, Teresa D. Lewis, Nelli Schetle, Thierry M. Work, Julie Dagenais, George H. Balazs, Jo-Ann C. Leong

**Affiliations:** 1 Institute of Virology, University of Zurich, Zurich, Switzerland; 2 BACPAC Resources Center, Children's Hospital Oakland Research Institute, Oakland, California, United States of America; 3 US Geological Survey, National Wildlife Health Center Honolulu Field Station, Honolulu, Hawaii, United States of America; 4 National Marine Fisheries Service, Pacific Islands Fisheries Science Center, Honolulu, Hawaii, United States of America; 5 Hawaii Institute of Marine Biology, University of Hawaii at Manoa, Kaneohe, Hawaii, United States of America; University of Kansas Medical Center, United States of America

## Abstract

The Chelonid fibropapilloma-associated herpesvirus (CFPHV; ChHV5) is believed to be the causative agent of fibropapillomatosis (FP), a neoplastic disease of marine turtles. While clinical signs and pathology of FP are well known, research on ChHV5 has been impeded because no cell culture system for its propagation exists. We have cloned a BAC containing ChHV5 in pTARBAC2.1 and determined its nucleotide sequence. Accordingly, ChHV5 has a type D genome and its predominant gene order is typical for the *varicellovirus* genus within the *alphaherpesvirinae*. However, at least four genes that are atypical for an alphaherpesvirus genome were also detected, i.e. two members of the C-type lectin-like domain superfamily (F-lec1, F-lec2), an orthologue to the mouse cytomegalovirus M04 (F-M04) and a viral sialyltransferase (F-sial). Four lines of evidence suggest that these atypical genes are truly part of the ChHV5 genome: (1) the pTARBAC insertion interrupted the UL52 ORF, leaving parts of the gene to either side of the insertion and suggesting that an intact molecule had been cloned. (2) Using FP-associated UL52 (F-UL52) as an anchor and the BAC-derived sequences as a means to generate primers, overlapping PCR was performed with tumor-derived DNA as template, which confirmed the presence of the same stretch of “atypical” DNA in independent FP cases. (3) Pyrosequencing of DNA from independent tumors did not reveal previously undetected viral sequences, suggesting that no apparent loss of viral sequence had happened due to the cloning strategy. (4) The simultaneous presence of previously known ChHV5 sequences and F-sial as well as F-M04 sequences was also confirmed in geographically distinct Australian cases of FP. Finally, transcripts of F-sial and F-M04 but not transcripts of lytic viral genes were detected in tumors from Hawaiian FP-cases. Therefore, we suggest that F-sial and F-M04 may play a role in FP pathogenesis.

## Introduction

The Chelonid fibropapilloma-associated herpesvirus (CFPHV; Chelonid herpesvirus 5, ChHV5) is strongly associated with fibropapillomatosis (FP), a neoplastic disease of marine turtles [1]. Based on previous partial sequencing of its genome, ChHV5 has been taxonomically assigned to the subfamily *alphaherpesvirinae* [1], [2], [3], [4]. FP was first described in the 1930s in green sea turtles (*Chelonia mydas*) from Florida [5]. Since then, FP has reached epizootic proportions, affecting at least five more marine turtle species worldwide, including the east and west coast of both Northern and Southern America, the Caribbean, Australia, Asia, and the Hawaiian Islands (reviewed in [6], [7]). In Florida, the prevalence of FP ranges from 11 to 52% [8]. FP is characterized by the formation of fibroepithelial tumors growing in the skin, in periorbital and orbital tissues, and in Hawaii, the oral cavity [7], [9]. Furthermore, tumors may grow in multiple visceral sites, including lung, liver, kidney, heart and gastrointestinal tract [10]. Tumor size may vary from 0.1 to more than 30 cm in diameter [10]. As a result of indirect effects of tumor growth, affected turtles become emaciated, bacteremic, immunosuppressed, and anemic [11], [12]. However, severe debilitation has also been observed with turtles carrying only few external or internal tumors [10]. Histologically, skin tumors are characterized as fibropapillomas [13], whereas internal tumors are characterized as fibromas, myxofibromas or fibrosarcomas of low grade malignancy [10]. All these tumor-types are ChHV5 DNA-positive and have extensive collagen deposition in the extracellular matrix with myxofibromas having deposition of sulfated proteoglycans between collagen fibers [10]. At present, it remains unclear, whether these alterations to the extracellular matrix are being caused by altered metabolism of the fibroblasts by transformation or directly by the function of unidentified viral enzymes that may be secreted from infected cells. Rarely, amphophilic intranuclear inclusion bodies, compatible with herpesvirus, are noted in the epidermal layers of tumors [14] suggesting that viral replication is restricted to certain areas of the tumor and, probably, to certain cell types. Furthermore, perivascular mononuclear cell infiltration has been observed frequently in the dermal layers of tumors [13].

While descriptions of the clinical signs, pathology, and pathogenesis of FP are numerous, research on the FP-associated herpesvirus itself has been impeded by the fact that no cell culture system for propagation of ChHV5 exists [15] and only a fraction of its genome has been sequenced; all known sequences confining to the putative Unique Long (UL) fragment of the genome [1], [3], [4], [7]. Koch's postulates have not yet been fulfilled to identify conclusively ChHV5 as the one and only causative agent of FP, although transmission of FP by using cell-free tumor material has been reported [16]. Having access to more sequence information about ChHV5 would be valuable in that it may open the doors for elucidation of molecular mechanisms of pathogenesis as well as for the development of diagnostic tests, antiviral treatments, and vaccines.

The purpose of the research described here was to (1) generate a Bacterial Artificial Chromosome (BAC) [17], [18], [19] of ChHV5 and (2) expand the knowledge on ChHV5 genomic sequences [1]. Indeed, we found that the ChHV5 genome is largely collinear with the genomes of typical alphaherpesviruses but that is also comprises a number of unexpected features.

## Results

### Identification a BAC containing known ChHV5 sequences

A BAC library was created from the glottis tumor of a Hawaiian green turtle with FP as described in Materials and Methods. With an estimated average insert size of 130 kbp (not counting the pTARBAC sequences), the library covered approximately two turtle genome sizes. Previously published sequences of ChHV5 (AF035003 [3], AY395516 [4], AF355149, AF355148, EF555201, AY644454 [1]) were used to generate three different hybridization probes for screening within the library for clones containing ChHV5 sequences. As a result, probes generated from UL30, UL22, and UL12 identified one single positive clone, which was designated CH-651-60O9. Presence of additional known ChHV5 sequences on the same clone was also confirmed by PCR, where primers for amplification of fragments from UL28 and UL10 were used (Table 1). A shotgun library was established from the BAC and subjected to DNA sequencing. The resulting sequences suggested that BAC CH-651-60O9 most probably contained the entire genome of ChHV5.

**Table 1:** Oligonucleotides used for identification of BAC CH-651-60O9.

Designation	oligonucleotide sequence (5′ to 3′)
UL10-F^a^	GCTAACATAAAGGCCGCGTA
UL10-R^a^	TGGCCTTTGGTGTTTACCTC
UL27F^a^	ATGATCATCGTCCTGTTA
UL27R^a^	GACCTGAGAATACGTCGA
UL28-F^a^	ATTCGTTCGACGTTTTCTGC
UL28-R^a^	TTGGACGAGGACCTTTCGTA
5′-pol^b^	ACTGGCTGGCACTCAGGAAA
3′-pol^b^	CAGCTGCTGCTTGTCCAAAA
pol-probe^c^	[6FAM]-CGATGAAAACCGCACCGAGCGA-[TAMRA]
UL12-OVa^d^	CGTGCTTAGGGTTCAAAAACACGG
UL12-OVb^d^	TGCGCTTTTCAGGTACCCGTGTTT
^32^P-dsUL12 probe^e^	CGTGCTTAGGGTTCAAAAACACGGGTACCTGAAAAGCGCA
UL22-OVa^d^	TGGCATCAGTAGGAGATAACGGAC
UL22-OVb^d^	TTCGGCTCTTTCGCAAGTCCGTTA
^32^P-dsUL22 probe^e^	TGGCATCAGTAGGAGATAACGGACTTGCGAAAGAGCCGAA
UL30-OVa^d^	TTTGGTGGACCAGACAACCTATCG
UL30-OVb^d^	ACATGGCGCAACAAACCGATAGGT
^32^P-dsUL30 probe^e^	TTTGGTGGACCAGACAACCTATCGGTTTGTTGCGCCATGT

a = PCR primers (F = forward; R = reverse)

b = Taqman primers

c = Taqman probe

d = oligonucleotide for hybridization (overlapping variants a and b were used to generate ^32^P-labeled probes

e = double stranded probe

### Sequence of BAC CH-651-60O9

**Figure 1:**
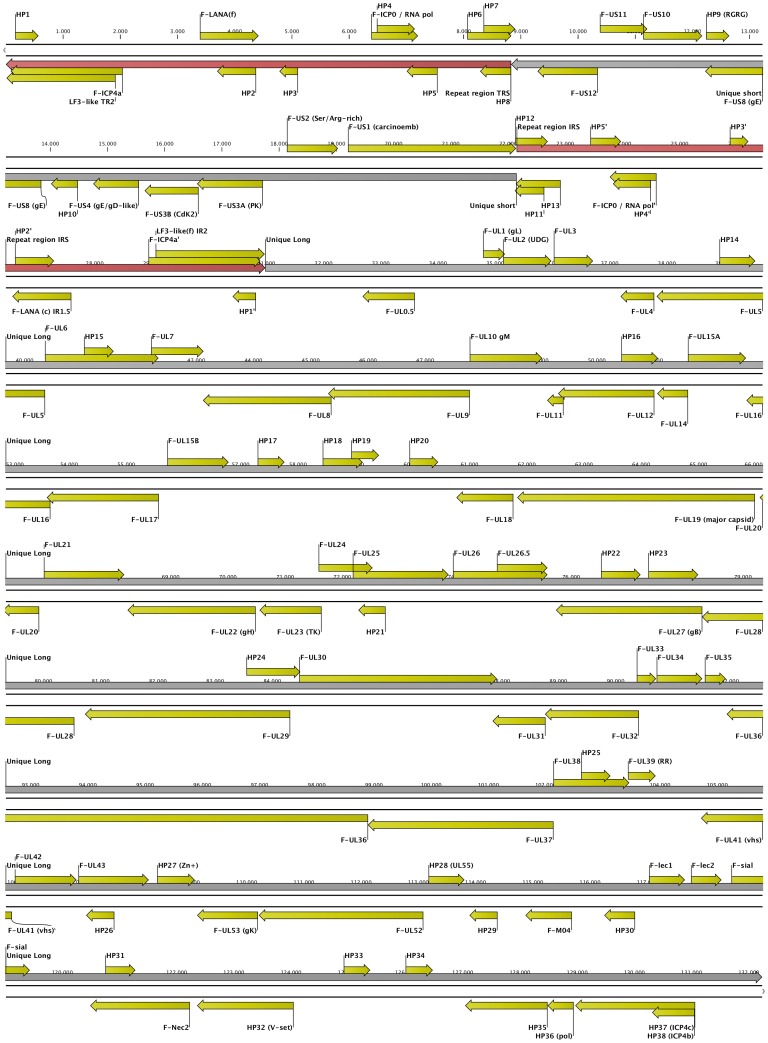
Genomic Map of ChHV5 as determined from BAC CH-651-60O9. The linearized, double stranded map, drawn to scale, shows the Repeat Sequences (TRS and IRS) in brown, the Unique Sequences (US and UL) in grey, and the predicted open reading frames (ORFs) in yellow. The relative orientation of each ORF is symbolized in the direction of its arrow shape. The ORF designations and the scale refer to the information provided in Tables 2 through [Table pone-0046623-t003] 4.

Apart of the pTARBAC sequences, BAC CH-651-60O9 comprised a total of 132.233 bp of DNA, which could be divided into a unique long sequence (UL; 101.152 bp), a unique short sequence (US; 13.319 bp), and inverted repeat sequences (IRS; 8.831 bp each), which flanked the US sequence. Thus, the cloned ChHV5 sequences presented themselves in the configuration of a herpesvirus Type D genome, such as it is found in the genus *varicellovirus* of the *alphaherpesvirinae* (Figure 1). The entire viral sequence was deposited as GenBank HQ878327. Since the entire sequence had been derived from a circular molecule, the numbering of the nucleotides was pragmatically started at one of the repeats and continued over US to the second (thus internal) repeat sequence, and finally along the UL sequence. This order was also maintained to briefly describe below some of the most interesting features of the sequence. Open reading frames (ORFs) that demonstrated by BLAST analysis a recognizable similarity to known herpesvirus proteins were named according to the current herpesvirus gene nomenclature and assigned with the prefix “F” for fibropapilloma.

### Repeats

Analysis of the repeated sequences revealed 12 ORFs with more than 40 codons, which encoded for 15 different potential proteins (Table 2). Each 6 ORFs were oriented in the same direction. Three loci consisted of overlapping ORFs. Three translated ORFs showed restricted homology to proteins encoded in the repeats of other herpesviruses. One of them showed a remote similarity to ICP0, the second exhibited a limited resemblance to ICP4. Both of these exhibited a signature sequence that has been associated to nucleotidylylation of the corresponding proteins [20]. The third one showed, interestingly, some alikeness to the latency-associated nuclear antigen (LANA) encoded in gammaherpesvirus genomes. Four ORFs (HP6, HP7, HP11, HP13) extend into the US sequence, thus, encoding for two pairs of putative proteins with identical N-termini and differing C-termini. One ORF (HP12) started from within the US, whereas its counterpart (HP8) was contained within the repeats but shared C-terminal identity with HP12.

**Table 2:** ORFs in the inverted repeats and their predicted features.

Designation	Map position	Predicted Features
HP1/HP1′	F(163..570)/C(26563..27591)	Secretory pathway; transmembrane region
F-LANA/F-LANA′	F(3391..4416)/C(26563..27591)	Nuclear localization; N-terminal DNA-binding domain
F-ICP0/F-ICP0′	F(6387..7202)/C(23780..24595)	Similar to TAF9 RNA polymerase II; Nucleotidylylation signature
HP4/HP4′	F(6484..7146)/C(23836..24498)	Hypothetical protein; ORF overlapping with F-ICP0
HP6/HP13	F(8063..8833)/C(22137..22919)	Nuclear localization; ORFs extending into US; N-terminally identical proteins
HP7/HP11	F(8349..8900)/C(22118..22633)	Nuclear localization; ORFs overlapping with HP6/13 and extending into US; N-terminally identical proteins
LF3-like/LF3-like′	C(12..1916)/F(29066..30970)	Leucine Zipper motif; C-termianl RNA-binding domain; similar to LF3 of Cercopithecine herpesvirus 15; nuclear localization;
F-ICP4a/F-ICP4a′	C(86..2041)/F(28941..30896)	Nuclear localization; remote similarity to ICP4; Nucleotidylylation signature
HP2/HP2′	C(3693..4373)/F(26609..27289)	Similarity to germ line helicase; overlapping F-LANA on the opposite strand
HP3/HP3′	C(4782..5105)/F(25877..26200)	Putative membrane protein; transmembrane helix: aa 33–55
HP5/HP5′	C(7007..7543)/F(23439..23975	Secretory pathway; transmembrane region: aa 10–27
HP8/HP12	C(8289..8831)/F(22127..22693)	Nuclear localization; products identical within C-terminus

### Unique Short sequence

Eleven ORFs were identified within the US sequence, 5 in one direction and 6 in the opposite direction (Table 3). Several of the predicted proteins did not show close relationship to known proteins of other herpesviruses. However, there were homologs to US10, US8 and US3 of other herpesviruses. The F-US10 protein was predicted according to its similarity to the virion protein US10 of a waterbird herpesvirus (Anatid herpesvirus 1). The protein encoded by F-US8 is apparently related to several glycoprotein E (gE) variants of different herpesviruses, including equine herpesvirus 9 as well as bovine and phocid herpesviruses. Interestingly, two different genes in the US of ChHV5 showed a relatively strong homology to the herpesvirus protein kinase US3; they were designated F-US3A and F-US3B. Moreover, the gene designated F-US4 encodes for a glycoprotein that shares a conserved C-11n-C-10n-C motif with the gG, gD, gI family of glycoproteins mapping in the US of various herpesviruses [21].

**Table 3 pone-0046623-t003:** ORFs in the Unique Short sequence and their predicted features.

Designation	Map position	Predicted Features
F-US11	F(10382..11209)	Nuclear localization
F-US10	F(11140..12168)	Similarity to virion protein US10 of Anatid herpesvirus 1
HP9	F(12238..12642)	RGRG Proteasomal degradation domain
F-US2	F(18137..19027)	Ser/Arg-rich protein
F-US1	F(19204..22140)	Secretory pathway; Signal sequence; transmembrane region
F-US12	C(9293..10345)	Nuclear localization; N-terminal RNA-binding domain; similarity to RNA-binding splicing factor
F-US8	C(12223..13842)	Glycoprotein E (gE); signal sequence; transmembrane region
HP10	C(14015..14482)	Nuclear localization; leuzine-rich
F-US4	C(14752..15549)	Glycoprotein; signal sequence; transmembrane region; IG_like domain; a
F-US3B	C(15650..16591)	Similarity to US3 serine/threonine protein kinase US3 (PK) and to cyclin-dependent kinase 2
F-US3A	C(16566..17714)	Similarity to US3 PK and catalytic domain of the Protein Serine/Threonine Kinase

Although none of the predicted proteins showed direct homology to the immediate early proteins 22 or 27, several candidates were detected, for which nuclear localization was predicted. For example, an N-terminal RNA-binding domain and similarity to RNA-binding splicing factor was predicted for F-US12, which might, therefore, contribute similar functions as either ICP27 or ICP22 of other alphaherpesviruses.

### Unique long sequence

The UL sequence was mostly collinear to the genomes of typical alphaherpesviruses. 76 ORFs were identified within the UL sequence, 40 in the forward direction and 36 in the opposite direction (Table 4). 46 ORFs showed strong similarities to UL genes of other herpesviruses. However, homologs to some UL-ORFs of other herpesviruses were amiss, i.e. UL13, UL40, UL44 through 51, and UL54 through 56. Interestingly, each one of those “missing genes” has been reported to be non-essential for viral viability in one or the other comparable herpesvirus [22]. In compensation for those missing genes, at least four genes were detected that are not typically associated with alphaherpesviruses but may be found in beta- or gammaherpesviruses. Moreover, 26 hypothetical genes were identified.

**Table 4:** ORFs in the Unique Long sequence and their predicted features.

Designation	Map position	Predicted features
F-UL0.5	c(32683..33594)	Putative nuclear protein
F-UL01	34789..35169	Glycoprotein L (gL)
F-UL02	35138..35980	Uracil DNA glycosylase (UDG)
F-UL03	36025..36708	Nuclear phosphoprotein
F-UL04	c(37192..37779)	Nuclear protein
F-UL05	c(37823..40357)	Putative component of DNA helicase/primase complex; helicase signature
HP14	38921..39544	Similarity to hypothetical protein of Gallid herpesvirus 1
F-UL06	40356..42338	Putative capsid portal protein
HP15	41039..41554	Hypothetical nuclear protein
F-UL07	42211..43125	Herpes_UL7 superfamily
F-UL08	c(43118..45361)	Putative UL8 Herpesvirus DNA helicase/primase complex associated protein
F-UL09	c(45306..47780)	Putative Origin-binding protein; Domain: DEAD-like helicases superfamily; ATP binding site on conserved domain DEXDc; putative Mg++ binding site on conserved domain DEXDc.
F-UL10	47779..49044	Glycoprotein M (gM); 99% identical to AAU84515.1 in GenBank
F-UL11	c(49134..49418)	Putative myristylated protein
F-UL12	c(49325..51004)	99% identity with gb|AAU84516.1| UL12 [Fibropapilloma-associated turtle herpesvirus]; YqaJ-like viral recombinase domain; herpesvirus alkaline exonuclease
HP16	50429..51067	Hypothetical protein; predicted bipartite NLS
F-UL14	c(51058..51594)	96% identity with gb|AAU84517.1| UL14 [Fibropapilloma-associated turtle herpesvirus]
F-UL15A	51593..52606	Probable DNA packing protein, N-terminus; 97% identity with gb|AAU84518.1| UL15A [Fibropapilloma-associated turtle herpesvirus]
F-UL16	c(52618..53673)	97% identical to gb|AAU84520.1| UL16 [Fibropapilloma-associated turtle herpesvirus]; herpesvirus UL16/UL94 family; capsid maturation, DNA packaging/cleavage.
F-UL17	c(53613..55568)	DNA packaging tegument protein; Multi domain PFAM: pfam04559, herpesvirus UL17 protein; 96% identity to gb|AAU84521.1| UL17 [Fibropapilloma-associated turtle herpesvirus]
F-UL15B	55718..56788	Probable DNA packing protein, C-terminus; 99% identity to gb|AAU84519.1| UL15B [Fibropapilloma-associated turtle herpesvirus]
HP17	57297..57764	Hypothetical protein
HP18	58434..59129	Unknown Protein; Similarity to protein RL5A [Human herpesvirus 5] gb|AAO48775.1|
HP19	58933..59415	Hypothetical protein; E value: 0.035: gb|AAR31234.1| protein RL6 [Human herpesvirus 5]
HP20	59950..60450	Hypothetical protein; E value 0.017: U54 HHV6B; ref|NP_050235.1| Gene info linked to NP_050235.1 virion transactivator [Human herpesvirus 6]
F-UL18	c(60772..61764)	99% identity to UL18 [Fibropapilloma-associated turtle herpesvirus] gb|AAU84522.1|; VP23 “capsid triplex subunit 2”
F-UL19	c(61835..65986)	Major capsid protein; 99% identity to gb|AAU84523.1| major capsid protein [Fibropapilloma-associated turtle herpesvirus]
F-UL20	c(66072..66701)	100% identical with gb|AAU84524.1| UL20 [Fibropapilloma-associated turtle herpesvirus]; Herpesvirus egress protein UL20; 5 TM helices predicted
F-UL21	66784..68187	85% identity with gb|AAU84525.1| UL21 tegument protein [Fibropapilloma-associated turtle herpesvirus]
F-UL22	c(68248..70485)	Putative glycoprotein H (gH); 98% identity to gb|AAU84526.1| glycoprotein H [Fibropapilloma-associated turtle herpesvirus]; predicted features: signal sequence; 4 N-gly sites; transmembrane helix
F-UL23	c(70555..71637)	98% identity to gb|AAU84527.1| thymidine kinase [Fibropapilloma-associated turtle herpesvirus]; C-terminal extension relative to gb|AAU93321.1| thymidine kinase [Hawaiian green turtle herpesvirus]
F-UL24	71586..72527	PFAM: cl03293 [Superfamily] cl03293, Herpes virus protein UL24; 100% identity with gb|AAU93322.1| membrane-associated protein [Hawaiian green turtle herpesvirus]; 98% identity with gb|AAU84528.1| UL24 [Fibropapilloma-associated turtle herpesvirus]
F-UL25	72184..73857	99% identity with gb|AAU93323.1| minor capsid protein [Hawaiian green turtle herpesvirus]; 98% identity with gb|AAU84529.1| UL25 [Fibropapilloma-associated turtle herpesvirus]
HP21	c(72282..72755)	Hypothetical protein; predicted NLS
F-UL26	73935..75584	Putative UL26 capsid maturation protease"; 100% identity with gb|AAU93324.1| capsid maturation protease [Hawaiian green turtle herpesvirus]; 99% identity with gb|AAU84530.1| UL26 [Fibropapilloma-associated turtle herpesvirus]
F-UL26.5	74703..75584	Putative UL26.5 virion scaffolding protein; 100% identity with gb|AAU93325.1| virion scaffolding protein [Hawaiian green turtle herpesvirus]
F-UL27	c(75736..78291)	Glycoprotein B (gB); 100% identity with gb|AAU93326.1| virion membrane glycoprotein B [Hawaiian green turtle herpesvirus]; 99% identity with gb|AAU84531.1| glycoprotein B [Fibropapilloma-associated turtle herpesvirus]; predicted features: signal peptide; 3 transmembrane helices
HP22	76522..77211	Hypothetical protein
HP23	77350..78219	Limited similarity to C3 Complement C3 precursor protein
F-UL28	c(78288..80540)	ICP18.5; 99% identity with gb|AAU93327.1| DNA cleavage/packaging protein [Hawaiian green turtle herpesvirus]
F-UL29	c(80728..84312)	Putative UL29 single-stranded DNA binding protein [Green turtle herpesvirus]; [Superfamily] cl09516, ssDNA binding protein; 99% identity with gb|AAQ67362.1| single-stranded DNA binding protein [Green turtle herpesvirus]
HP24	83548..84489	6-phosphofructokinase-like hypothetical protein; NLS predicted
F-UL30	84473..87925	Putative DNA polymerase catalytic subunit [Hawaiian green turtle herpesvirus]; 98% identity with gb|AAU84534.1| polymerase [Fibropapilloma-associated turtle herpesvirus]
F-UL31	c(87852..88775)	[Superfamily] cl14325, nuclear egress lamina protein UL31; 99% identity with gb|AAU93329.1| nuclear phosphoprotein [Hawaiian green turtle herpesvirus]
F-UL32	c(88768..90408)	100% C-terminal identity with gb|AAU93330.1| DNA cleavage/packaging protein [Hawaiian green turtle herpesvirus
F-UL33	90371..90706	DNA cleavage/packaging protein [Hawaiian green turtle herpesvirus] (Quackenbush et al., Virology 246 (2), 392-399 (1998); 100% identity with gb|AAU93331.1| DNA cleavage/packaging protein [Hawaiian green turtle herpesvirus]
F-UL34	90719..91513	100% identity with gb|AAU93332.1| UL34 membrane-associated phosphoprotein [Hawaiian green turtle herpesvirus]
F-UL35	91561..91926	100% identity with gb|AAU93333.1| VP26 basic phosphorylated capsid protein [Hawaiian green turtle herpesvirus]
F-UL36	c(91944..98897)	PFAM: [Superfamily] cl04174, Herpesvirus UL36 VP1/2 tegument protein; This family only covers a small central part of this large protein.
F-UL37	c(98897..102139)	PFAM: [Superfamily] cl04350, Herpesvirus UL37 tegument protein; closest similarity to gb|AAY59063.1| small tegument protein [Tortoise herpesvirus]
F-UL38	102138..103463	PFAM: [Superfamily] cl04010, Herpesvirus capsid shell protein VP19C; closest similarity to gb|AAY59064.1| minor capsid protein [Tortoise herpesvirus]
HP25	102623..103132	Hypothetical protein with predicted bipartite NLS
F-UL39	103441..103923	Partial similarity to gb|AAQ73541.2| ribonucleotide reductase large subunit [Tortoise herpesvirus]
F-UL41	c(104719..105897)	Closest similarity to gb|AER28066.1| tegument host shutoff protein [Gallid herpesvirus 1]; PFAM: cd09867, PIN domain of Flap Endonuclease-1, a structure-specific, divalent-metal-ion dependent, 5′ nuclease and homologs.
F-UL42	105949..107025	DNA polymerase processivity factor; similarity to ref|NP_944415.1| DNA polymerase processivity subunit [Psittacid herpesvirus 1];
F-UL43	107061..108287	Similarity to gb|AEI00251.1| UL43 protein [Gallid herpesvirus 3]
HP26	c(107196..107687)	Hypothetical protein
HP27	108437..109090	Hypothetical protein of 217 aa with at least two Zink-binding domains identified; similarity to ref|YP_001285929.1| protein IG [Psittacid herpesvirus 1]
F-UL53	c(109136..110197)	PFAM: [Superfamily] cl03284, UL53 cell fusion glycoprotein K; highest blast scores with envelope glycoprotein gK gK of psittacid herpesvirus 1, anatid-, felid1, gallid3, equid4 herpesviruses; 6 transmembrane helices predicted
F-UL52	c(110214..113087)	PFAM: [PHA03180], UL52 helicase-primase primase subunit; similarity to gb|AAG30093.1|AF282130_56 DNA helicase/primase complex protein [Meleagrid herpesvirus 1]
HP28 (UL55)	113179..113805	Limited similarity to UL55 on aa level; E-value 93–04 similarity to ref|YP_443903.1| nuclear protein UL55 [Papiine herpesvirus 2]
HP29	c(113896..114384)	Hypothetical protein
F-M04	c(114874..115683)	Similarity to emb|CAJ84726.1| m04 protein [Murid herpesvirus 1]; m04 proteins are known to mediate immune evasion by interference with the major histocompatibility complex class I (MHC-I) pathway of antigen presentation to cytolytic T lymphocytes. Instead, the m04 early gene product binds to folded MHC-I molecules in the ER and directs the complex to the cell surface; two transmembrane helices predicted
HP30	c(116252..116788)	Hypothetical protein; ORF may N-terminally extend for up to 46 aa; similarity to emb|CAC85009.1| hypothetical protein [Saimiriine herpesvirus 2]; predicted features: signal sequence, transmembrane helix; N-glycosylation site at pos. 40 NFTL
F-lec1	117032..117658	Probable start codon GGC; PFAM: cl02432 [Superfamily] cl02432, C-type lectin (CTL)/C-type lectin-like (CTLD) domain; CLECT: C-type lectin (CTL)/C-type lectin-like (CTLD) domain; PFAM: cd03593, C-type lectin-like domain (CTLD) of the type found in natural killer cell receptors (NKRs); CLECT_NK_receptors_like: C-type lectin-like domain (CTLD) of the type found in natural killer cell receptors (NKRs); transmembrane helix predicted
F-lec2	117767..118297	Alternative AAA-start codon at position −57; similar to type II membrane protein CD69 [Equus caballus]; E-value: 5e-09: “C-type lectin domain family (poxviruses)”; PFAM: cd03593, C-type lectin-like domain (CTLD) of the type found in natural killer cell receptors (NKRs); CLECT_NK_receptors_like: C-type lectin-like domain (CTLD) of the type found in natural killer cell receptors (NKRs); E value 1e-10: dbj|BAG69470.1| c-type lectin-like receptor [Gallus gallus]; transmembrane helix predicted
F-sial	118474..119433	Similarity to CMP-N-acetylneuraminate-beta-galactosamide-alpha-2,3-sialyltransferase (mus musculus); PFAM: cl02965 [Superfamily] cl02965, Glycosyltransferase family 29 (sialyltransferase) ; E value 6e-70: emb|CAB53395.1| Gal(beta)1,3/4-GlcNAc (alpha)2,3-sialyltransferase [Mesocricetus auratus]; transmembrane helix predicted
F-Nec2	c(120491..122230)	Similarity to CD155 (poliovirus receptor) and CD112 (Nectin-2); E-value: 0.016 with glycoside hydrolase family 5 [Eubacterium cellulosolvens 6]; signal sequence predicted
HP31	120755..121279	Hypothetical protein with similarity to Tau-tubulin kinase 1; E-value: 0.034
HP32	c(122359..124044)	Hypothetical V-set protein; pfam07686, Immunoglobulin V-set domain; Signal sequence predicted
HP33	124921..125382	Hypothetical protein
HP34	126003..126476	Hypothetical protein
HP35	c(127044..128483)	PFAM: cl02885 [Superfamily] cl02885, heptad repeat 1-heptad repeat 2 region (ectodomain) of the transmembrane subunit of various endogenous retroviruses (ERVs) and infectious retroviruses; signal sequence and transmembrane helix predicted
HP36	c(128486..128938)	Hypothetical protein; PFAM: [Superfamily] cl11403, Cellular and retroviral pepsin-like aspartate proteases.
HP37 (ICP4c)	c(128969..131062)	Hypothetical protein with limited similarity to ICP4 stretching over several domains; predicted features: several NLS, DNA- and RNA-binding domains
HP38 (ICP4b)	c(130312..131055)	Hypothetical protein with similarity to ICP4s over several regions; predicted features: C-terminal RNA-binding domain; mitochondrial targeting peptide

### Are the newly detected unconventional genes truly part of the viral genome?

**Figure 2:**
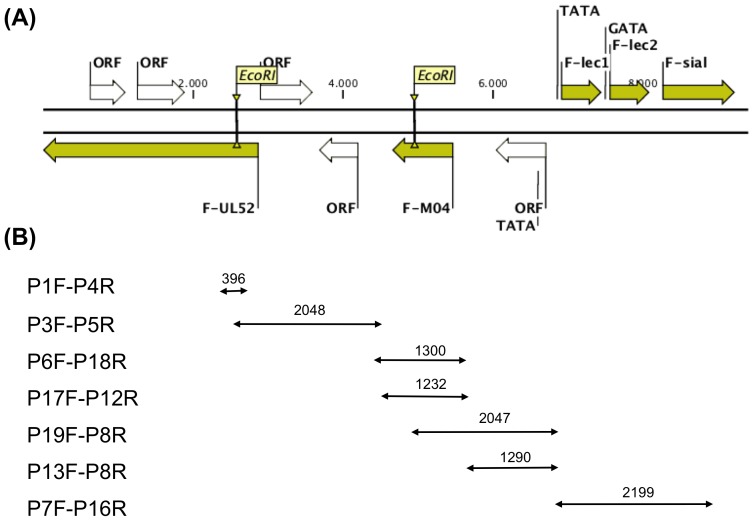
Map of atypical ChHV5 sequences and strategy for overlapping PCR. (A) A double stranded map of the atypical ChHV5 sequences is shown, anchoring in F-UL52. The arrows indicate relative length and orientation of each ORF; yellow ORFs were object of further analysis in this study; numbers refer to the nucleotide count in the forward orientation. EcoRI sites as well as predicted TATA- and GATA-boxes are indicated. The pTARBAC vector in BAC CH-651-60O9 (not shown) was integrated at the EcoRI site within F-UL52. (B) PCR amplification using the primer pairs (see Table 5) listed on the left was expected to yield the products listed on the right. Double-arrows indicate the putative map location of the PCR product; the numbers refer to the expected sizes of the amplicons.

According to the sequence determination, the pTARBAC sequences had inserted into an EcoRI site within the F-UL52 open reading frame. With parts of UL52 flanking the insertion, it was assumed that the BAC comprised the entire viral genome. However, while conventional UL genes extended on one side of the UL52 ORF, we detected on the other side of the same gene a row of ORFs that were rather unexpected in the context of an alphaherpesvirus genome (Table 4; Figure 2A). The first one was similar to cytomegalovirus M04 (F-M04, 270 aa), the second and third one showed similarity to the C-type lectin domain family (F-lec1, 178 aa; F-lec2, 176 aa), and the fourth one carried the signatures of a sialyltransferase (F-sial, 320 aa). Thus, the question arose, whether or not the newly detected unconventional genes were truly part of the ChHV5 genome.

**Table 5:** PCR primers used for overlapping PCR.

Designation	oligonucleotide sequence (5′ to 3′)
P1F^a^	CGACATGCCCCAAATAATGTCG
P2R^a^	GAATTCGTGCCTCTTGGGTAACC
P3F	AATTCAGTAAGCATTGTATTGT
P4R	GACCTGGGCTACTTGATAAGGG
P5R	CATTTTTATTAAGGAGGAAACC
P6F	TTAGATCGCCAGTTTGGTTA
P7F	CATCAGCCTTTGCGTATTT
P8R	CCTTAACGCTCGAAACAATA
P12R	CATCAGACTCCAAAACCAAA
P13F	GAGGAAAGATAGGCGCAAA
P16R	GGGGACCACTTTGTACAAGAAAGCTGGGTT**TACGTTAGGCAGACGTTC** b
P17F	GGTTTCCTCCTTAATAAAAATG
P18R	TTTGCGCCTATCTTTCCTC
P19F	AGGTAACAGGTTTTGCGAAG

a = PCR primers (F = forward; R = reverse)

b = this primer consists of a binding part (bold) and is fused to a 5′-att-recombination sequence. The latter part of the primer was not required for the present experiment but was, nevertheless, part of the primer.

**Figure 3:**
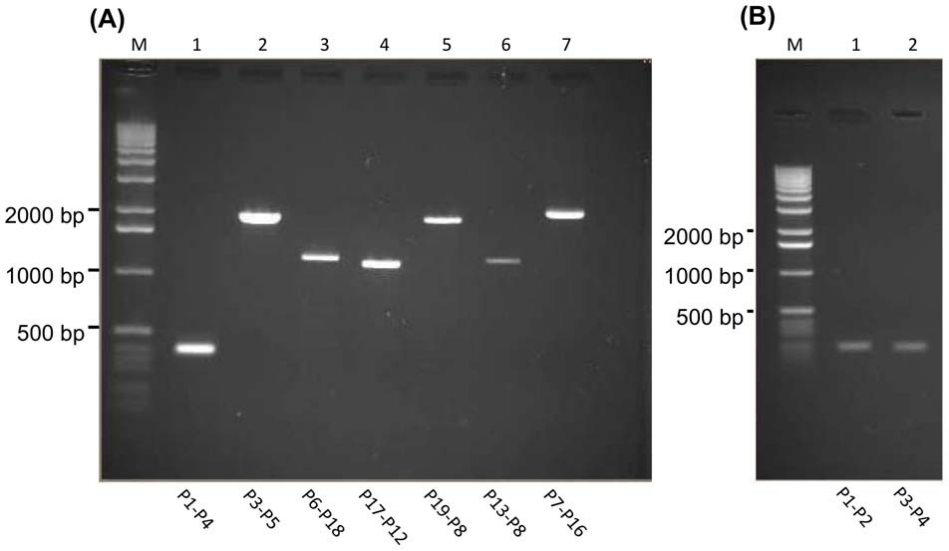
Overlapping PCR. Ethidium bromide stained agarose gels with the PCR products are shown. (A) PCR products generated with DNA from FP-related tumors as template. The primer pairs used for amplification of each product are indicated below the corresponding lane; M: size marker with specific band sizes provided at the left. For expected sizes of the amplification products in lanes 1 through 7, see Figure 2B. (B) amplification products obtained with primer pairs P1–P2 (201 bp) and P3–P4 (198 bp), when BAC CH-651-60O9 DNA was used as template.

In a first approach to address this, overlapping PCR, starting from within UL52, was performed. DNA from tumor materials of independent FP-cases was used as template, while BAC CH-651-60O9 DNA and DNA from unaffected tissue of the same turtles served as controls. The primers used are given in Table 5, while the PCR-strategy together with the expected map locations and sizes of the amplification products are indicated in Figure 2B. The primer pair P1F and P4R gave a product of the expected 396 bp size solely when DNA extracted from tumors was used as template (Figure 3A). Due to the pTARBAC insert, the amplification product size with the BAC DNA template would have been larger than 10.000 bp, which exceeded the limits of our PCR conditions and did not result in a visible product (not shown). Due to lack of template, no PCR product arose when DNA from normal tissue was used (not shown). However, the other primer pairs gave products of the expected sizes with either BAC CH-651-60O9 DNA (not shown) or DNA extracted from tumors (Figure 3A) but not with DNA from normal tissue (not shown). To confirm the suitability of the primers P1F and P4R in the context of our BAC DNA, they were paired with P2R and P3F, respectively, which provided amplification products of the expected size with either DNA extracted from tumors (not shown) or BAC CH-651-60O9 DNA (Figure 3B) but not with DNA from normal tissue as template (not shown).

Together, these data strongly suggest that these unconventional genes are actually part of the ChHV5 genome. They also support the notion that the BAC CH-651-60O9 comprises the entire genomic DNA of ChHV5.

### The F-sial- and the F-M04 genes are expressed in tumor tissue

**Figure 4:**
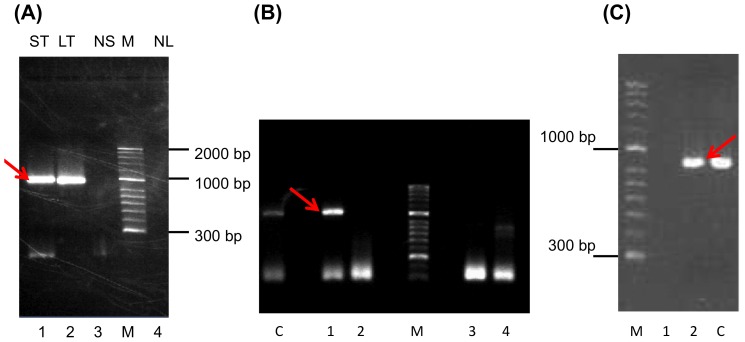
RT-PCR for detection of F-sial- and F-M04 RNA. RNA extracted from fresh tumors as well as from corresponding normal tissue was subjected to RT-PCR as described in materials and methods and the amplification products were separated on a 1%agarose gel. (**A**) **F-sial**: Lane 1, skin tumor (ST); lane 2, lung tumor (LT); lane 3, normal skin (NS); lane 4, normal lung (NL). M, HyperLadder II was used as a molecular weight standard. The red arrow points to the expected 960 bp RT-PRC product. (**B**) **Control reactions** were performed to demonstrate that RNA was a true template. Lane C, to confirm the DNA polymerase activity of the enzyme used in reactions 1 through 4, DNA template was used and the RT step was omitted. Lane 1, RNA extracted from a skin tumor was reverse transcribed and subjected to PCR; lane 2, same as lane 1 but the RT step was omitted; lanes 3 and 4, same as lanes 1 and 2 but the template stemmed from normal skin. M, HyperLadder II. Note: a faint unspecific product may be detected in Lane 4, which is not of the expected size. The red arrow points to the expected 960 bp RT-PRC product. (**C**) **F-M04**: M, HyperLadder II. Lane 1, RNA extracted from a skin tumor; RT-step omitted; PCR using F-M04 primers. Lane 2, same as lane 1 but RT step was executed. Lane C, DNA template was used and the RT step was omitted. The red arrow points to the expected 869 bp RT-PCR product.

Tumor as well as matching normal tissue from fresh cases of FP was collected and RNA was extracted for RT-PCR as described in materials and methods. The primers used for this experiment, designed to target either the F-sial- or the F-M04-gene, are listed in Table 6. Using this approach, the corresponding RNAs for both F-sial (Figure 4A, B) and F-M04 (Figure 4C) were detected in different tumors but not in the corresponding normal tissue that had been collected just outside of the tumor area of the same animal. Upon omission of the RT step, no product was generated, whereas the polymerase used was shown to amplify DNA template (Figure 4B, C). Consecutively, the amplification products were cloned by recombination into a Gateway donor vector and the nucleotide sequence of each insert was determined. Each sequence matched exactly the corresponding prediction (data not shown). In contrast to F-sial and F-M04, the question regarding transcription products for either F-UL52 or F-lec1 or F-lec2 was not addressed. However, various RNAs extracted from different tumors were also analyzed for the transcripts for the envelope glycoprotein gB (F-UL27), the capsid protein VP26 (F-UL30), the capsid portal protein (F-UL6), and the viral thymidine kinase (F-UL23) all with negative result (data not shown). Thus, this series of experiments indicated that both F-sial and F-M04 were expressed in FP-tumors, while genes for components related to viral replication were not expressed. Moreover, the sequences of these two newly discovered unconventional viral genes matched exactly to the sequences determined from the BAC CH-651-60O9 DNA.

**Table 6:** PCR primers used for detection of F-M04- and F-sial RNA^a^.

Designation^b^	oligonucleotide sequence (5′ to 3′) c
Att-SiaF	GGGGACAAGTTTGTACAAAAAAGCAGGCT**ATGAGGCGCAATGTAAAA**
P16R	GGGGACCACTTTGTACAAGAAAGCTGGGTT**TACGTTAGGCAGACGTTC**
Att-M04F	GGGGACAAGTTTGTACAAAAAAGCAGGCT**ATGAAAAGATACTCGTTC**
M04R-att	GGGGACCACTTTGTACAAGAAAGCTGGGTT**TTGAATTAAATACAAATC**

a = these primers were also used to amplify and clone (for sequence determination) the Australian isotypes of F-M04 and F-sial

b = PCR primers (F = forward; R = reverse)

c = each primer consists of a binding part (bold) and is fused to a 5′-att-recombination sequence. The latter part of the primer was used for consecutive cloning and sequencing of the amplification product into a Gateway donor vector.

## Discussion

While it is almost standard procedure to clone BACs from replication competent herpesviruses, this is, to our knowledge, the first time that a genomic herpesvirus BAC has been created directly from infected tissue and in the absence of any means to propagate the agent in culture [7], [18], [19], [23], [24], [25]. Total DNA was extracted from PCR-positive turtle tumors, partially digested, size selected and cloned into a BAC vector [26]. One out of 18.000 BAC clones was identified as carrying known ChHV5 sequences, including hybridization by using probes against UL30, UL22, and UL12 as well as by PCR using primers for detection of UL28, UL27 (gB), and UL10 (data not shown).

The nucleotide sequence determination of the BAC revealed that the overall structure of the cloned molecule corresponded in its size (132.233 bp) and configuration (TRS-US-IRS-UL) to a Type D genome, which is typical for members of the *varicellovirus* genus of the *alphaherpesvirinae*. Indeed, the largest part of the molecule encoded for proteins that are known to be typical for those viruses (Tables 2, 3, 4). The sequences of several fragments of the ChHV5 UL sequence were previously deposited by others and us in GenBank and were available for comparison, revealing conservation in the range of 98 to 100%. In addition, corresponding homologues were easily detected by BLAST analysis. However, 38 potential coding sequences were also found that did not readily provide an alphaherpesvirus-related counterpart upon BLAST analysis. With a few exceptions (addressed further below), those ORFs were designated as potentially coding for hypothetical proteins (HP). The sequence analysis also revealed that pTARBAC had indeed inserted into the UL 52 gene of ChHV5 (F-UL52), a gene that belongs to the helicase/primase complex, which is essential for replication of herpesviruses [27], [28].

### Comparison of the predicted F-UL52 protein to its herpesvirus orthologs

A P-BLAST search of the predicted F-UL52 translation product suggested a relationship to UL52 proteins from various animal alpha herpesviruses, including equid (EHV1), bovid (BoHV1), suid (SuHV1), melagrid (MeHV1), and gallid (GaHV1, 2, 3) herpesviruses. A more distant relationship to beta herpesviruses was also revealed, for example to human (HCMV) and chimpanzee (PaHV2) cytomegaloviruses. Upon alignment and tree-formation of the UL52 aa sequences using the neighbor joining algorithm, the newly discovered F-UL52 species emerged on the same branch as bovid and suid alphaherpesviruses and cytomegaloviruses whereas avian alphaherpesviruses clustered on a different branch (Figure S1).

Upon pairwise comparison of the individual aa sequences (Table S1), the level of aa identity among the different herpesvirus UL52 sequences varied mostly around 31 to 43% identity. Highest identities (82%) were found in between of the closely related bovid herpesviruses 1 and 5, followed by the avian herpesviruses (∼60%), F-UL52 (22 and 25%) and betaherpesvirus (7 to 10%).

There is considerable variation in aa length of UL52 of herpesviruses. Alphaherpesvirus UL52s range from 962 to 1124 aa, whereas Betaherpesviruses have much smaller UL52 homologs (640 to 668 aa). With a predicted size of 957 aa, the F-UL52 was closer in size to the UL52s of the alphaherpesviruses and may explain in part the topology of the UL52 phylogenetic tree (Figure S1). Interestingly, we occasionally obtained larger than expected PCR products with primers P1F and P4R, which we attribute to repeat sequences within the targeted F-UL52 gene, although size variation might also occur in F-UL52 of different ChHV5 isolates (data not shown).

In summary, F-UL52 has a distant but distinct relationship to UL52 of the *alphaherpesvirinae* subfamily but does not have an especially close relationship to avian UL52. Since the gene itself extended to both sides of the pTARBAC-insertion, the possibility that a fragment of the ChHV5 genome had been lost due to the cloning procedure may be regarded as diminished, although not entirely excluded.

### Unique short sequence and its flanking repeats

To our knowledge, this is the first time that sequences extending to the US fragment of ChHV5 and its flanking repeats are reported. Two very interesting features were observed in the sequence of the ChHV5 US fragment. First, two individual genes (F-US3A and F-US3B), sharing absolutely no apparent sequence homology between each other, were detected, both of which appeared to encode for a herpesvirus protein kinase. Conventionally, the US3 gene encodes for a protein kinase (PK) that is characteristic for the *Alphaherpesvirinae* subfamily of the herpesviruses [29]. However, the US3 PK is known to fulfill various functions, which may include effects on the cytoskeleton of the infected cell, anti-apoptotic activity, and function(s) in the egress of viral particles [29], [30], [31]. It is tempting to speculate that each one of these two newly discovered PKs may fulfill separate functions; one that might be important for viral replication, the other (anti-apoptotic) that may play a role in the pathogenesis of tumor formation. The total lack of sequence homology between F-US3A and F-US3B, on both the nucleotide and the amino acid sequence level, speaks against a possible emergence of the two genes due to a gene duplication mechanism. The second interesting feature of ChHV5 US relates to the number of encoded glycoprotein genes. In herpes simplex virus type 1 (HSV-1), the US sequence harbors a row of 5 genes, all in the same orientation, which encode for viral glycoproteins (US4 gG, US5 gJ, US6 gD, US7 gI, US8 gE). However, in ChHV5 US we detected only two such genes, designated here F-US4 and F-US8. By BLAST analysis, F-US8 was readily identified as a gE-homologue. In contrast, the F-US4 protein only remotely resembled to herpesvirus glycoproteins. It is well known that members of the genus varizellovirus, such as its prototype member varizella-zoster virus (VZV) or the Bovine herpesviruses types 1 and 5 (BoHV1, BoHV5) may feature a lesser number of glycoprotein genes in their US sequence [32], [33], [34], [35]. Importantly, at least one of the encoded proteins supplies the major receptor-binding function. In most alphaherpesviruses, this property is provided by gD (US6 protein). In VZV, the protein encoded by gene 68 (corresponding to US8, gE) has evolved to execute this function in the absence of an US6 (gD) homolog. In the absence of a ChHV5 that replicates in cell culture, it is presently difficult to address the question, which one of the two ChHV5-US-encoded glycoproteins actually confers the receptor-binding function. Since the receptor-binding protein needs to interact with the virus's fusion machinery, consisting of gB, gH, and gL, it is doubtful that a simple transfer of the candidate receptor-binding proteins to another gD-deficient alphaherpesvirus will be able to identify the corresponding property. However, such an approach has been successful in the case of the two very closely related viruses BoHV1 and BoHV5 [36]. Based on the presence of a conserved C-11n-C-10n-C motif present in gG, gD, and gI, others have speculated that the corresponding glycoprotein genes may have evolved through processes of duplication and subsequent divergence [21]. Since this exact motif is apparently conserved in the F-US4-encoded protein, it may be speculated that it may indeed represent a progenitor form of the gG, gD, gI family of modern alphaherpesviruses.

While in our sequence determination the TRS and IRS sequences *per se* were perfectly mirrored, they provided limited similarity to repeat sequences known from other alphaherpesviruses. The largest ORF within those repeats typically encodes for a member of the ICP4 family, an immediate early regulator of viral gene expression. Indeed, such an ORF was detected among the ChHV5 sequences (F-ICP4a), whose translation product carried a nuclear localization signal and also exhibited the consensus nucleotidylylation sequence that has been annotated to ICP4 [20]. Both features are consistent with a potential functional homolog to ICP4, although proof will have to be provided by future experiments. Interestingly, the F-ICP4a ORF was overlapped in an alternative frame with an even slightly larger coding sequence, which exhibited similarity to the LF3 gene of Cercopithecine herpesvirus 15 (therefore designated LF3-like). It will be interesting to find out, whether only one or the two of these ORFs are functional in ChHV5 replication. Alternatively, it may also be that F-ICP4a is part of a spliced transcription product, which includes other coding sequences. Candidates for such processes were detected in the UL sequence (designated HP38 ICP4b and HP37 ICP4c). However, those genes would only be available for transcription starting from the F-ICP4a gene if the viral genome would circularize. Alternative splicing of transcripts starting from within the repeated sequences and extending into UL, thus giving rise to a product (*circ*) that may be produced only upon circularization of the genome, has been reported for example in BoHV1 [37].

### Atypical genes

Interestingly, a series of genes was present in our BAC-cloned DNA molecule that may be atypical for alphaherpesviruses but each one of them has well defined homologues in the genomes of beta- or gammaherpesviruses. None of these gene products is known to have an essential role in viral replication. However, each one apparently plays a biological relevant role in either pathogenesis or immunedeviation. One of these (F-M04), has only been described in beta herpesviruses, while another (F-sial) has been found in a gammaherpesvirus (BoHV4), in other virus families like poxviruses and baculoviruses, and also in host cells [38]. Orthologues to F-lec1 and F-lec-2, respectively, have been detected in the genome of rat CMV as well as several cytoplasmic DNA viruses, i.e. poxviruses and asfarviruses [39], [40], [41].

Overall, these observations suggest that ChHV5 may combine features of not only the *alphaherpesvirinae* but also of *beta*- and *gammaherpesvirinae*. Recent analyses of the available genomic sequences of reptilian herpesviruses suggested that ChHV5 as well as the lung-eye-trachea disease-associated herpesvirus of green turtles (ChHV6) [42], [43] should be counted among the alphaherpesviruses [2], [44], [45]. Interestingly, the existence of atypical genes has been reported to occur among the mardiviruses, which comprise tumorigenic avian alphaherpesviruses [2], [45], [46], [47]. It is therefore interesting to note that ChHV5 shares the property of harboring host-related genes not only with the beta- and gammaherpesviruses but also with the mardiviruses.

### Features of the predicted F-lec proteins

According to the predicted amino acid (aa) sequences both of these putative proteins are type II membrane proteins and carry the signature of the C-type lectin-like domain superfamily (reviewed in [48]). The superfamily consists of at least 16 different groups. Group V, which seems to be most close to the present F-lec proteins, comprises relatively small (∼20 kDa), non-calcium-binding type II membrane proteins, which are associated with either activation or inhibition of natural killer (NK) cells. Most of those proteins have protein ligands but some are also multivalent, binding not only proteins but also carbohydrates. The C-type lectin protein orthologue in rat CMV (designated RCMV lectin) originates from a multiple-spliced gene [41]. In contrast, the poxviruses (e.g. vaccinia virus A40R) and ASFV (8CR) carry unspliced variants of a similar gene [39], [40]. Upon alignment of these viral lectins with F-lec1 and F-lec2 as well as the equine CD69 protein (as a presumed outlier) only very limited similarity was observed among the individual aa sequences. Indeed, upon pairwise comparison, the percentage of aa identity varied between 12 to 23% (Table S2). Surprisingly, the highest identity percentages of F-lec1 and F-lec2 were neither detected among the two themselves (19.8% aa identity) nor among the other viral orthologs (12 to 15%) but with the CD69-like protein of the horse (*equus caballus*)(22% and 23%). Unfortunately, the corresponding sequences from *Chelonia mydas*, the virus's host, are presently not known.

To our knowledge, ChHV5 is the first virus detected to carry at least two separate genes coding for this type of host-like proteins. Notably, a TATA box was observed in the presumed promoter region of F-lec1 (less than 50 bp upstream of the ATG). In contrast, the F-lec2 gene was preceded with a GATA box in its presumed promoter region. Interestingly, the GATA box had been reported as a functional feature from within the promoter of the RCMV lectin gene [41].

### Features of the predicted F-M04 protein

According to the predicted aa sequence, the putative ChHV5 M04 (F-M04) protein is a type I membrane protein with signal sequence (aa 1–24), an extracellular domain (aa 25–230, a transmembrane region (aa 231–253), and a cytoplasmic tail (aa 254–270). Furthermore, at least one N-glycosylation site (N53) and an O-glycosylation site (S37) can be predicted on its ectodomain. Furthermore, a motif-scan revealed a COX2 domain (aa 54–63), an Ig- and MHC-signature (aa 180-186), and a BPD-transp-1-domain (aa 227–262; Binding-protein-dependent transport system inner membrane component). Many of those features are typically found on M04 proteins. Moreover, the predicted size of F-M04 (270 aa) matches nicely into the typical M04 size pattern, which ranges from 256 aa to 271 aa. However, alignment of F-M04 aa sequence (Table S3) with members of the M04 family specified by various murid cytomegaloviruses revealed only a minor relationship. MCMV members of the M04 family showed among each other an identity level of approximately 40 to 90%. In contrast, F-M04 shared between 13 and 18% identity with other members of the family.

In MCMV, M04 has been shown to associate with MHC-I molecules and to travel in their company to the cell surface, where the complex may serve as a decoy for preventing effective NK activity [49], [50]. However, the M04 gene, which belongs to the m02 family of MCMV genes, is subject to considerable variation in nature, which might affect the function of its corresponding gene product [51]. It will be most interesting to test in the future how this applies for the product of F-M04.

### Features of the predicted F-sial protein

According to the predicted aa sequence, the putative F-sial (Figure S2) is a type II membrane protein, comprised of 320 aa, with a transmembrane region close to its *N*-terminus (aa 7–26) and with extensive similarity to the conserved domains of any typical sialyltransferase, i.e. domain *L* (aa 105–152), *S* (aa 246–268, III (aa 281–284), and VS (aa 296–301) [52], [53].

Sialyltransferases are important in viral pathogenesis (reviewed in [38]). Alignment of the amino acid sequence of F-sial with selected members of the protein family (Table S4) revealed three pairs that were clearly related to each other, i.e. (1) the acetylglucosamine transferases of Bovine herpesvirus 4 (BoHV4) and its host (*Bos taurus*)(94% aa identity), (2) the sialyltransferases of myxoma virus and rabbit fibroma virus (79%), and (3) the sialyltransferases IV of humans (*Homo sapiens*) and chicken (*Gallus gallus*) (75%). Interestingly, the protein specified by Deerpox virus shared 39% identity with the proteins of the other poxviruses, whereas F-sial was closest to the human and chicken proteins (36% identity) but shared still about 27% identity with the poxvirus proteins, which also shared >30% identity with the human and chicken proteins. All other proteins analyzed shared only 12% or less identity among each other and with F-sial. The stretches of highest homology were found in the central region of the molecules as well as towards the C-terminus, i.e. in the context of the proteins' predicted functional domains.

Viral sialyltransferases have been reported to glycosylate not only proteins (poxviruses and bovine gamma herpesvirus 4, BoHV-4) but also ecdysteroid hormones (baculoviruses) and DNA (bacteriophages). In each of those examples, the viral glycoslytransferase seems to play an important biological role. While bacteriophages are able to switch their serotype as a result of the viral glycosyltransferase activity, insect molting, and pupation is inhibited because of the baculovirus encoded glycosyltransferase. The only herpesvirus that is currently known to encode a sialyltransferase, BoHV-4, apparently gains a great survival advantage by possessing this gene in its repertoire. Descendents from this relatively modern virus are found worldwide, whereas the glycosyltransferase-less progenitor has been extinguished [38], [54], [55]. Fascinatingly, myxomavirus and other leporipoxviruses that possess sialyltransferases are able to give rise to the growth of localized fibromas in rabbits, hares, and squirrels [56]. Although the respective sialyltransferases are not exclusively responsible for the fibromas, they are considered as important virulence factors of the corresponding viruses. The morphological similarity between those syndromes and FP suggests that parallels in the underlying pathogeneses may exist.

### Are the unconventional genes truly part of the ChHV5 genome?

One may argue that our approach to generate the ChHV5 BAC clone is tainted with undesirable drawbacks. Due to restriction enzyme digestion and ligation, fragment(s) of the viral genome may be lost and non-viral sequences may be integrated into the final construct. Depending on the site of the pTARBAC insertion, there is also the possibility that the emerging BAC clone may not be infectious. Indeed, this third *caveat* actually came true: pTARBAC was inserted inside of the coding sequences of F-UL52, which encodes for the viral helicase that is essential for viral replication. Thus, infectious virus could not be reconstituted from our BAC (data not shown). Yet, this deficiency may be corrected throughout future experiments. The present sequence knowledge provides ample information to plan for the transfer of the pTARBAC cassette to a more desirable location. However, the insertion inside of a coding sequence with parts of the interrupted gene to either side of the insert has also its advantages for the present purpose. Indeed, it argues strongly against the potential loss of viral sequences that might have occurred during the cloning process. Moreover, it contributes a first line of evidence that the unconventional genes are actually part of the viral genome. A second line of evidence came from overlapping PCR using DNA from unrelated cases of FP as template and starting the series of PCR from the far side of the pTARBAC-insertion locus. Moreover, none of the unconventional sequences were amplified, when the DNA template was extracted from unaffected tissue of the same animals. A third line of evidence was contributed by the results of 454 pyrosequencing DNA extracts from independent tumors and normal skin (data not shown). Importantly, no additional herpesvirus-like sequences were detected, while the previously determined ChHV5 sequences were confirmed, including those of the unconventional genes. Finally, DNA from several FP-tumors of Australian origin (kindly provided by Dres. Graham Burgess and Ellen Ariel) was tested by PCR. The presence of F-UL27, F-M04, and F-sial sequences in these extracts was confirmed (data not shown). Yet, the Australian UL27 nucleotide sequence differed from our Hawaiian sequence by a characteristic CGACTC-insert that has been identified previously [4]. Moreover, sequence analysis of the amplification products obtained from the Australian templates revealed at least two consistent base chances in the F-M04 gene and three within the F-sial gene (data not shown).

In conclusion, we provide new sequence information about ChHV5, which likely covers the entire viral genome. Interestingly, a series of genes was detected throughout this work that are very uncommon for a candidate member of the alphaherpesviruses. While transcripts of selected viral genes that are known to be active during herpesvirus replication were not detected in RNA extracts from tumor tissue, at least two of the newly detected ChHV5 genes, i.e. F-M04 and F-sial, were not merely present but rather expressed in the tumors. This observation suggests that they indeed may play a role in the pathogenesis of FP. Due to their predicted biological activities, it may be speculated F-sial might be involved in tumor formation, whereas F-M04 might protect the infected cells from NK cell activity. The latter notion might imply that MHC-I presentation is also affected in the tumors, which then would interfere with the successful development of anti-tumor vaccines.

## Materials and Methods

### Samples from green sea turtle and nucleic acid extraction

All samples were taken post mortem from animals with terminal FP. DNA for BAC cloning was extracted from a glottis tumor of a green turtle (*Chelonia mydas*) that stranded in October 2003 on Oahu, Hawaii. Additional DNA was extracted from skin tumors of three green turtles from Oahu that also stranded there in October 2003. DNA for overlapping PCR as well as RNA extracts originated from tissues (normal skin and skin tumor, normal lung and lung tumor, normal heart and heart tumor) of a green turtle on 30 May 2007. DNA was extracted using Qiagen genomic tips (Qiagen, Valencia, CA) according to standard procedures and stored at −80°C until further use. For RNA extraction, samples of non-tumored tissues and corresponding tumors were cut into small pieces, ground and homogenized in RTL solution (Qiagen) before being extracted using the Qiagen RNeasy kit from (Qiagen). RNA was resuspended in DEPC-treated water and stored at −80°C until further use.

### Detection of ChHV5 DNA

For the determination of the viral DNA load in turtle tissues, the method of Quackenbusch [57] was used without any modification. The primers and probes used are listed in Table 1.

### Generation of BAC library

A sample from the frozen glottis tumor of a green turtle was ground under liquid nitrogen using mortar and pestle, resuspended in CIB buffer (20 mM NaCl, 80 mM KCl, 15 mM Tris-HCl, 0.5 mM EGTA, 2 mM EDTA, 0.2 mM Spermine, 0.5 mM Spermidine, 18 mM β-Mercaptoethanol, pH 7.2) and homogenized using a Dounce homogenizer. The resulting cell and tissue suspension was mixed with 1% InCert agarose (Cambrex) for high molecular weight DNA extraction. The agarose embedded DNA was partially digested with a combination of EcoRI restriction enzyme and EcoRI methylase before being size fractionated by pulsed-field electrophoresis. DNA fragments from the appropriate size fraction were cloned into the pTARBAC2.1 vector [58] between the two EcoRI sites. The ligation products were transformed into DH10B (T1 resistant) electro-competent cells (Invitrogen) [26]. The library was arrayed into ninety-six 384-well microtiter dishes and subsequently gridded onto two 22×22 cm nylon high-density filters for screening by probe hybridization. Each hybridization membrane represented more than 18,000 distinct BAC clones, stamped in duplicate. Sample of random clones were digested with rare cutting NotI restriction enzyme (New England BioLabs) to estimate the average insert size.

### Identification of BAC CH-651-60O9

Overlapping oligonucleotides UL12-OVa and UL12-OVb (Table 1) were annealed to each other, ends were filled in with Klenow polymerase fragment, and ^32^P-ATP and ^32^P-CTP were added to the reaction to produce the 40-base pair ^32^P-labeled double stranded (ds) UL12-probe. Likewise, oligos UL22-OVa/OVb were used to produce ^32^P-dsUL22 probe whereas oligos UL30-OVa/OVb were used to produce ^32^P-dsUL30 probe. All three probes were purified separately on MicroSpin™ G-50 columns (Amersham), mixed, denatured, and hybridized to the nylon membranes with the BAC clones arrayed in duplicates. Two pairs of PCR primers (Table 1), UL10-F, UL10-R (expected product size 388 bp) and UL28-F, UL-28-R (expected product size 395 bp) were used to confirm presence of herpes DNA in clones identified as positive by hybridization.

### Sequencing of BAC CH-651-60O9

DNA sequencing was performed using the di-deoxy chain termination sequencing method on a shotgun library approach using pCR4bluntTopo (Invitrogen). Plasmid subclones were cycle-sequenced with Big-Dye terminator version 1.0 reagents (Applied Biosystems) and analyzed on a MegaBace 1000 sequencer (Amerham Biotech) or a ABI 377 sequencer (Applied Biosystems). Computer-assisted assembly was done with Lasergene SeqMan (DNASTAR Inc.).

### Overlapping PCR

Between 80 and 160 ng of DNA were used per PCR reaction. DNA was amplified with the primers listed in Table 3 in the presence of 10 nm dNTP, DMSO, 5x Phusion GC buffer and 2 U of Phusion polymerase (New England Biolabs, Ipswich, MA). For amplification, the samples were denatured at 98°C for 1 min, followed by 34 cycles at 98°C for 10 sec, 60° for 20 sec, 72°C for 50 sec, and a final extension at 72°C for 5 minutes. Synthetic oligonucleotides were obtained from Microsynth (Balgach, Switzerland).

### RT-PCR

Between 100 and 200 ng of RNA was used as template for the BioScript OneStep RT-PCR (Bioline, Staunton, MA). The RT-PCR mix contained the primers (Table 4) and 25 µl 2x OneStep buffer, 2 µl OneStep enzyme mix, 5 µl RNase inhibitor and DEPC-treated water up to a volume of 50 µl. After cDNA synthesis at 50°C for 60 min and inactivation of the RT-activity at 95°C for 10 min, the samples were subjected to the same PCR protocol as described above. Negative controls included DEPC-treated water without RNA and normal amounts of RNA but with the OneStep enzyme mix replaced by Phusion DNA polymerase, which was considered inactive for RT-activity. In a parallel reaction, the Phusion enzyme was used with a DNA template to control for its DNA polymerase activity.

## Supporting Information

Figure S1 **UL-52 alignment tree.** The CLC Main Workbench 5 software was used for this purpose. A pBLAST search, using the F-UL52 aa sequence for query, provided a list of viruses specifying related proteins. The viruses emerging on top of this list were used to create the progressive multiple alignments underlying this tree. Taxonomic status of these viruses and Swiss-Prot accession numbers: **Alphaherpesviruses**: Equid herpesvirus 1 (EHV1; P28962.1), Bovid herpesviruses 1 and 5 (BoHV1, Q65817; BoHV5, Q6X264), Suid herpesvirus 1 (SuHV1, pseudorabies virus, PRV, Q5PP97; SuHVK, Kaplan strain of PRV, Q85228), Cercopithecine herpesvirus 16 (CeHV16; also known as papiine herpesvirus 2 of the baboons, which belongs to the genus simplex virus, Q2QBC3), Infectious laryngotracheitis virus (ILTV; Iltovirus of chickens, Q9YZA1), Melagrid herpesvirus 1 (MeHV1; herpesvirus 1 of Turkeys, Q9DGY9), and Gallid herpesvirus type 1, (GaHV1, Q9IBS6), Gallid herpesvirus type 2 (GaHV2, Q9E6M4), and Gallid herpesvirus type 3 (GaHV3, Q782P1), Anatid herpesvirus 1 (AnHV1; alphaherpesvirus infecting ducks, geese and swans, B4XS04). **Betaherpesviruses:** Human cytomegalovirus (HCMV, A8T7D0) and Panine herpesvirus 2 (PaHV2; cytomegalovirus of the chimpanzee, Q8QS36).
(TIF)

Figure S2 **F-sial domains**. From N to C terminus the following domains of the predicted F-sial molecule are indicated: TM, transmembrane domain; L, L domain; S, S domain; III, domain III; VS, domain VS. In the lower part, the sequences of the conserved domains of F-sial are aligned with mouse- (GenBank accession BAA06068) and myxoma virus (GenBank accession U46577) sialyltransferases. Identical aa are boxed in black. Signature aa that are also highly conserved in other sialyltransferases are marked with an asterisk.
(TIF)

Table S1 F-UL52 pairwise alignment. The accession numbers for the sequences used are Swiss-Prot: P28962.1 (EHV1); Q6X264 (BoHV5); Q65817 (BoHV1); Q5PP97 (SuHV1); Q85228 (SuHV1, Kaplan strain); A8T7D0 (HCMV); Q8QS36 (PaHV2); Q2QBC3 (CeHV16); Q9YZA1 (ILTV); Q782P1 (GaHV3); Q9E6M4 (GaHV2); Q9IBS6 (GaHV1); Q9DGY9 (MeHV1); B4XS04 (AnHV1). Taxonomic information. **Alphaherpesviruses**: Equid herpesvirus 1 (EHV1), Bovid herpesviruses 1 and 5 (BoHV1, BoHV5), Suid herpesvirus 1 (SuHV1; pseudorabies virus, PRV; SuHVK represents the Kaplan strain of PRV), Cercopithecine herpesvirus 16 (CeHV16; also known as papiine herpesvirus 2 of the baboons, which belongs to the genus simplex virus), Infectious laryngotracheitis virus (ILTV; Iltovirus of chickens), Melagrid herpesvirus 1 (MeHV1; Herpesvirus 1 of Turkeys), and Gallid herpesviruses types 1, 2, and 3 (GaHV1, 2, 3; Herpesviruses of chickens), Anatid herpesvirus 1 (AnHV1; alphaherpesvirus infecting ducks, geese and swans). **Betaherpesviruses**: Human cytomegalovirus (HCMV) and Panine herpesvirus 2 (PaHV2; cytomegalovirus of the chimpanzee).
(XLSX)

Table S2 F-lec pairwise alignment. The accession numbers for the sequences used are: XM_001499388 (EqCD69); P24765(VV-A40); AF302184 (RCMV lectin); AAC28421 (ASFV-8CR). Abbreviations: Eq, equine, horse; VV, vaccinia virus, reference strain WR; RCMV, rat cytomegalovirus; ASFV, african swine fever virus.
(XLSX)

Table S3 F-M04 pairwise alignment. Swissprot accession numbers are indicated in the leftmost column. Regarding the variability of m04 in MCMV, see Corbett AJ et al. (2007). Extensive sequence variation exists among isolates of murine cytomegalovirus within members of the m02 family of genes. J Gen Virol 88: 758–769.
(XLSX)

Table S4 F-sial pairwise alignment. The accession numbers for the sequences used are: M35027 (Rabbit Fibroma virus); U46577.1 (Myxoma virus); NC_006967.1 (Deerpox virus); XM_417860.2 (Gallus gallus); NM_006278.1 (Homo sapiens); Q9IZK2 (BoHV4); Q7YQE1 (Bos taurus); M96361.1 (Baculovirus); A4ZUA5 Ampullavirus; A93469.1 (Fowl adenovirus).
(XLSX)
