# Supplementary figures and images for: The Genome of Chelonid Herpesvirus 5 Harbors Atypical Genes
Source: PLoS One. 2012 Oct 2;7(10):e46623. doi: 10.1371/journal.pone.0046623 (PMC3462797; doi:10.1371/journal.pone.0046623)

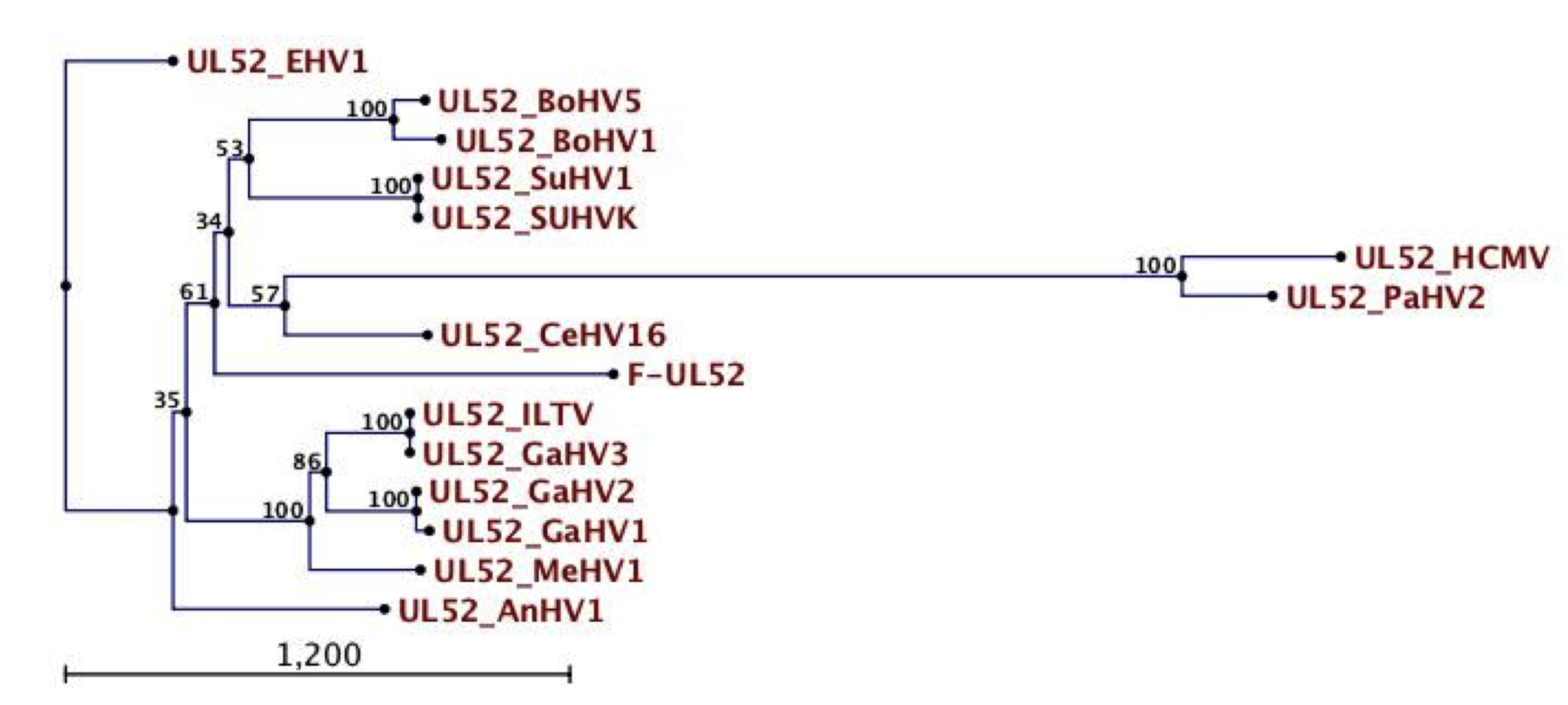

Supplement: Figure S1 — UL-52 alignment tree. The CLC Main Workbench 5 software was used for this purpose. A pBLAST search, using the F-UL52 aa sequence for query, provided a list of viruses specifying related proteins. The viruses emerging on top of this list were used to create the progressive multiple alignments underlying this tree. Taxonomic status of these viruses and Swiss-Prot accession numbers: Alphaherpesviruses: Equid herpesvirus 1 (EHV1; P28962.1), Bovid herpesviruses 1 and 5 (BoHV1, Q65817; BoHV5, Q6X264), Suid herpesvirus 1 (SuHV1, pseudorabies virus, PRV, Q5PP97; SuHVK, Kaplan strain of PRV, Q85228), Cercopithecine herpesvirus 16 (CeHV16; also known as papiine herpesvirus 2 of the baboons, which belongs to the genus simplex virus, Q2QBC3), Infectious laryngotracheitis virus (ILTV; Iltovirus of chickens, Q9YZA1), Melagrid herpesvirus 1 (MeHV1; herpesvirus 1 of Turkeys, Q9DGY9), and Gallid herpesvirus type 1, (GaHV1, Q9IBS6), Gallid herpesvirus type 2 (GaHV2, Q9E6M4), and Gallid herpesvirus type 3 (GaHV3, Q782P1), Anatid herpesvirus 1 (AnHV1; alphaherpesvirus infecting ducks, geese and swans, B4XS04). Betaherpesviruses: Human cytomegalovirus (HCMV, A8T7D0) and Panine herpesvirus 2 (PaHV2; cytomegalovirus of the chimpanzee, Q8QS36). (TIF) [file pone.0046623.s001.tif]

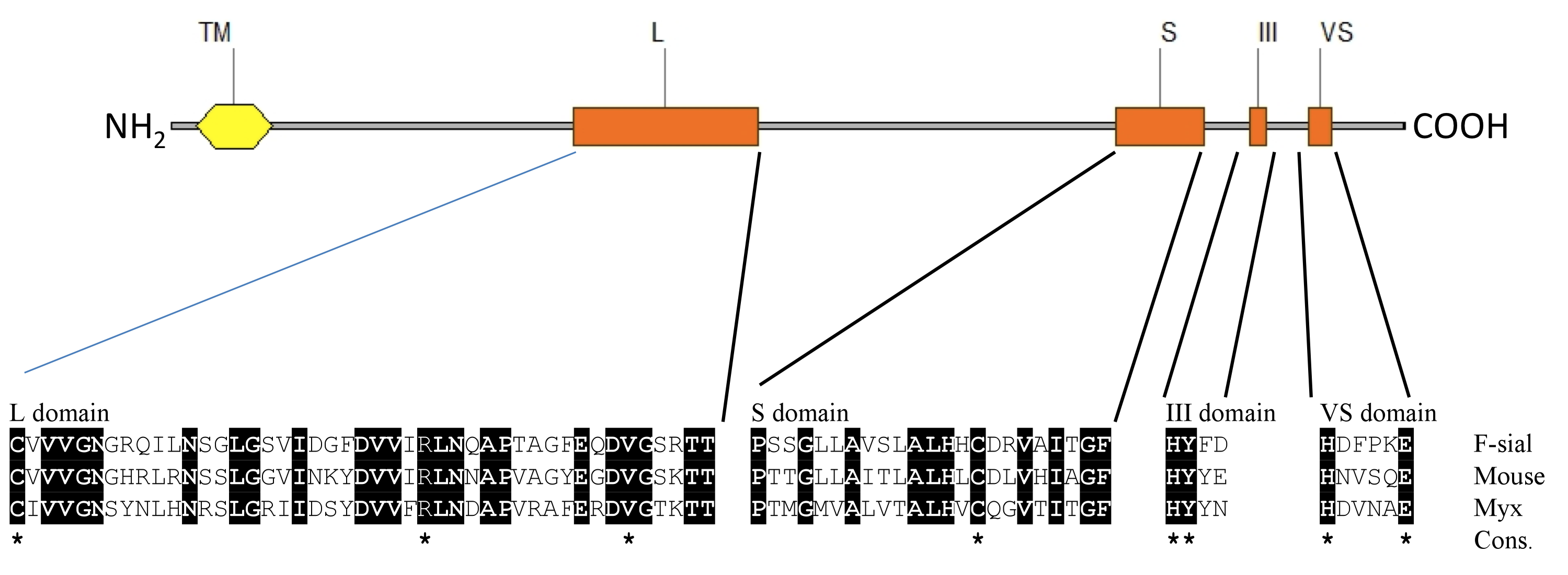

Supplement: Figure S2 — F-sial domains. From N to C terminus the following domains of the predicted F-sial molecule are indicated: TM, transmembrane domain; L, L domain; S, S domain; III, domain III; VS, domain VS. In the lower part, the sequences of the conserved domains of F-sial are aligned with mouse- (GenBank accession BAA06068) and myxoma virus (GenBank accession U46577) sialyltransferases. Identical aa are boxed in black. Signature aa that are also highly conserved in other sialyltransferases are marked with an asterisk. (TIF) [file pone.0046623.s002.tif]
